# Supplementary material for: Association between oral microbiome and seven types of cancers in East Asian population: a two-sample Mendelian randomization analysis
Source: Front Mol Biosci. 2023 Nov 21;10:1327893. doi: 10.3389/fmolb.2023.1327893 (PMC10702768; doi:10.3389/fmolb.2023.1327893)
Supplement: Supplementary file 1 [file DataSheet1.ZIP › Supplementary Materials/MR plots of saliva/saliva═╝/Lung Cancer/pheno.561_to_lung cancer_leave_one_out.pdf]

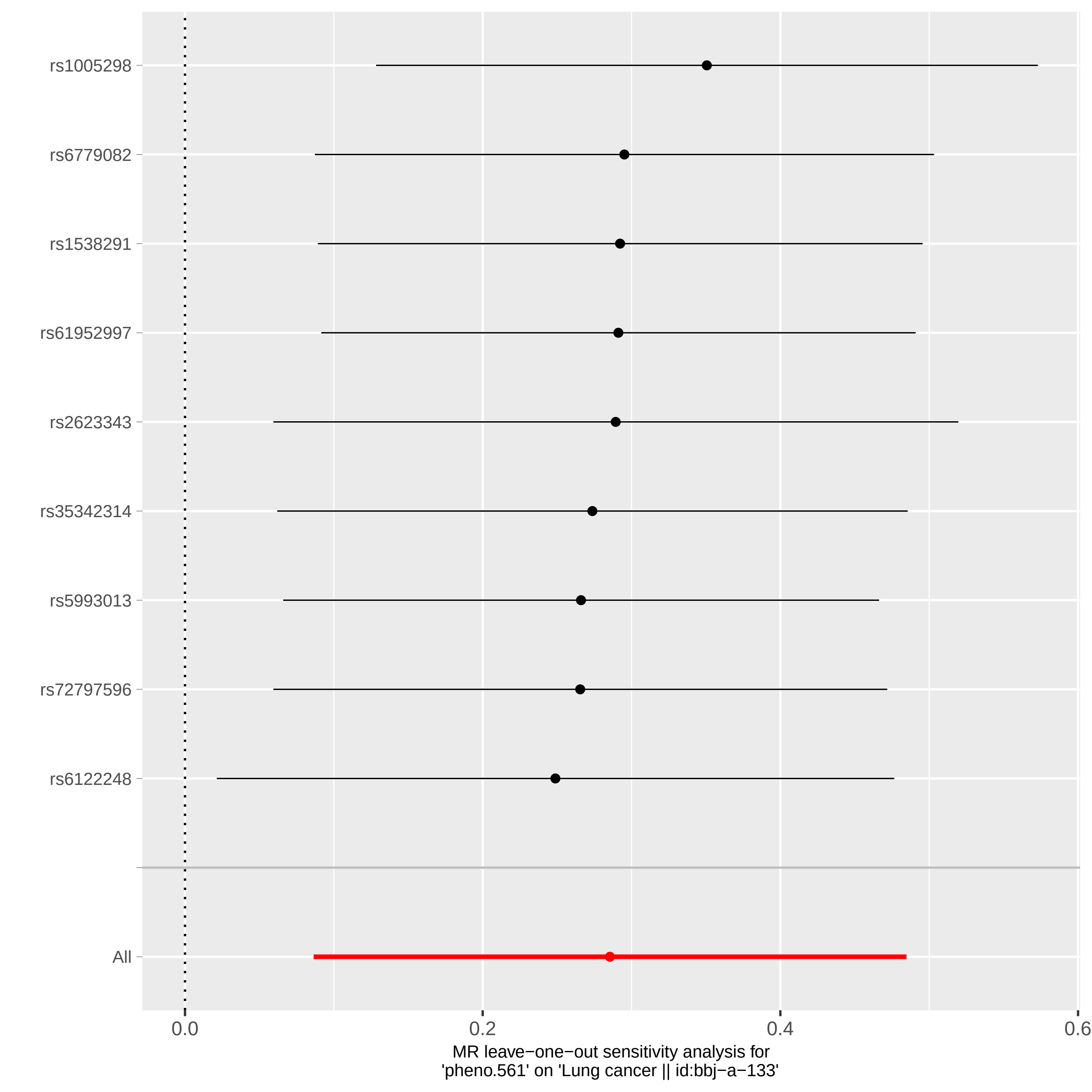
rs1005298
rs6779082
rs1538291
rs61952997
rs2623343
rs35342314
rs5993013
rs72797596
rs6122248
All
0.0
0.2
0.4
0.6
MR leave−one−out sensitivity analysis for
'pheno.561' on 'Lung cancer || id:bbj−a−133'
